# Anatomy as Applied to Oral Surgery and Block Anesthesia*Read before the American Society of Exodontists, Boston, Massachusetts, August 20, 21, 1920. Reprinted from *Journal N. D. A*., September.

**Published:** 1921-11

**Authors:** Arthur E. Smith

**Affiliations:** Chicago, Illinois


### ANATOMY AS APPLIED TO ORAL SURGERY AND BLOCK ANESTHESIA*

BY ARTHUR E. SMITH, D.D.S., M.D., CHICAGO, ILLINOIS

Every specialty in the professions of medicine and dentistry has for its foundation a thorough knoweldge of that broad subject, anatomy. Anatomy and chemistry are the two subjects a thorough understanding of which is necessary before the student can master the other subjects, which have anatomy as their foundation. Anatomy is divided, first, into microscopic and macroscopic, and, second, into several branches, namely, embryology, histology, biology, and gross anatomy.

A surgeon, physician, or dentist cannot practice good surgery, medicine, or dentistry without a knowledge of anatomy and chemistry any more than an individual can maintain good health without proper nourishment, fresh air, and exercise. They are the fundamental sciences.

---

* Read before the American Society of Exodontists, Boston, Massachusetts, August 20, 21, 1920. Reprinted from *Journal N. D. A.*, September.

Thorough training of the dental student in embryology, histology, pathology and gross anatomy is highly essential, and in many instances training in these branches has been overlooked or grossly neglected, but the student cannot be blamed entirely for the reason that some dental colleges have not given a complete course in these important subjects. It is a well-known fact that during the past the consensus of opinion among dental students was that they did not need to be familiar with embryology, histology, zoology, biology and gross anatomy. This erroneous idea still seems to lurk in the minds of some dental students.

The practitioner who has not obtained training in these fundamental sciences realizes today his sad mistake. He not only finds himself greatly handicapped in his private practice, but it is also utterly impossible for him to enter the great field of experimental research.

All professional research has for its foundation either anatomy or chemistry. It would not have been possible to know what we do today about the ravages and results of focal infection were it not for the knowledge we have of histology and pathology, both of which are based upon anatomy. It would not be possible to practice oral surgery and block anesthesia without a thorough familiarity of the anatomic parts. Surely an impacted third molar should not be removed or a radical operation for empyema of the antrum performed or a deep block injection made by an operator who was in doubt as to the anatomy. Gross anatomy teaches us the dependence and interdependence of parts, their location and relationship to one another, but an intelligent understanding of gross anatomy cannot be obtained without first knowing the primary subjects, embryology, histology and biology. It is indeed gratifying to note the advancement which has been made within recent years in many colleges in presenting these important branches in a more complete manner.

It matters not whether you are practicing oral surgery, orthodontia, prosthetic or operative dentistry, prophylaxis, pyorrhea, or the interpretation of the dental radiograph, a knowledge of anatomy is essential. Many errors in diagnosis have been made from a lack of knowledge of anatomy, not only in oral surgery, but in the interpretation of the radiograph. To be an efficient diagnostician and operator one needs to know the principles of anatomy. Many a misinterpretation of the radiograph has been caused by a lack of familiarity with the coronoid process, zygomatic arch, malar bone, mental foramen, inferior dental canal, antrum, unerupted or impacted teeth and varying densities of process and bone. The tissues of many patients have been ruthlessly mutilated in oral surgery and many block injections have been failures, because of the ignorance of technic and improper diagnosis.

The internal maxillary artery is seldom involved in oral operations, but its various branches are implicated to a considerable extent. These branches are highly important to the oral surgeon, inasmuch as they supply the operative field many times.

The inferior dental artery gives rise to considerable hemorrhage when the lower jaw is operated for such operations as fractures, alveolectomy, and the extraction of badly impacted lower third molars.

The deep temporal branch is involved and may give troublesome hemorrhage, unless ligated during the removal of the Gasserian ganglion on the severance of the sensory root of the fifth nerve for the treatment of tic douloureux, The infraorbital artery is also highly important to the oral surgeon for it is involved in operations upon the infraorbital nerve. The descending palatine artery passes downward and through the posterior palatine canal with the anterior palatine nerve from Meckel's ganglion, emerging upon the hard palate through the posterior palatine foramen. When operating for cleft palate this artery and its branches cause hemorrhages and in many instances ligation is very helpful.

The upper portion of the pharynx is supplied by the pterygo-palatine and Vidian branches which cause hemorrhage following the removal of adenoids. The spheno and descending palatine arteries supply the superior part of the tonsil and in many instances their severance results in serious hemorrhage following tonsillectomy. These small vessels give rise to considerable hemorrhage while operating on Meckel's ganglion. The nasopalatine artery passes downward and forward along the nasal septum and an operation upon the tissues forming the nasal septum may cause profuse hemorrhage, unless checked.

The posterior superior alveolar artery enters the posterior superior alveolar foramen (with the nerve of the same name) and supplies the alveolar procees, major portion of the antrum, buccal muco-periosteum, molars and bicuspid teeth in the upper jaw, and considerable hemorrhage follows an operation upon these tissues via this artery. During the resection of the upper jaw, hemorrhage will occur from a number of the branches of the internal maxillary artery. For a major operation involving the tissues in the region of the internal maxillary artery, it is good practice to ligate the internal carotid, which assists the operator to an immeasurable extent in checking hemorrhage.

When blocking the second division of the fifth nerve by the intraoral method or the blocking of the superior posterior alveolar nerve employing the correct technic, the needle does not come near the internal maxillary artery.

The nervus trigeminus and its connecting branches with other nerves is indeed difficult anatomy to master and understand. The location of this nerve with its extreme nerve complexity requires considerable study. No doubt you will agree with me that while you were a student in college that more time was spent on this part of anatomy than on any other part of the human body, and we realized our inability to discuss intelligently this nerve and all of its connecting links on examination day.

I realize that many dentists need to review this important subject before they attempt the practice of oral surgery, or make the deep block anesthesia injections. It would be wholly unwarranted and unjustified for any practitioner to subject his patient to deep block anesthesia injections if he is not acquainted with the nerve branches and their location.

The inserting of needles promiscuously into the tissues or the undertaking of a major oral operation if the anatomy involved is not understood is certainly bad practice. It is quite true that little knowledge of anatomy is necessary for making submucous injections, but for deep nerve-blocking injections, such as the blocking of the first, second and third divisions of the fifth nerve and their important branches, and the various branches from the cervical plexus and cervical ganglia, it is imperative that the operator must have his knowledge of anatomy at his finger tips in order to execute his work in an efficient manner. The successful oral surgeon has a clear mental picture of the anatomical parts involved and can easily outline them on paper or on the blackboard.

